# Effectiveness and Safety of Mitral Valve Plasty in Patients with an Anomalous Origin of the Coronary Artery from the Pulmonary Artery

**DOI:** 10.3390/jcdd10020075

**Published:** 2023-02-09

**Authors:** Lizhi Lv, Xinyue Lang, Simeng Zhang, Cheng Wang, Yuanhao Jin, Aihua Zhi, Qiang Wang

**Affiliations:** 1 Center for Pediatric Cardiac Surgery, Fuwai Hospital, National Center for Cardiovascular Diseases, Chinese Academy of Medical Sciences and Peking Union Medical College, Beijing 100037, China; 2 Department of Cardiac Surgery, Yunnan Fuwai Cardiovascular Hospital, Kunming 650102, China; 3 Medical Research & Biometrics Center, National Center for Cardiovascular Diseases, The National Clinical Research Center for Cardiovascular Diseases, Fuwai Hospital, Chinese Academy of Medical Sciences & Peking Union Medical College, Beijing 102308, China; 4 Department of Cardiac Surgery, Peking University People’s Hospital, Beijing 100044, China; 5 Department of Radiology, Fuwai Hospital, National Center for Cardiovascular Diseases, Chinese Academy of Medical Sciences and Peking Union Medical College, Beijing 100037, China; 6 Department of Radiology, Yunnan Fuwai Cardiovascular Hospital, Kunming 650102, China

**Keywords:** anomalous coronary artery from the pulmonary artery, anomalous origin of the left coronary artery from the pulmonary artery, total coronary artery from the pulmonary artery, simultaneous mitral valve plasty, mitral valve regurgitation

## Abstract

The study aimed to determine the effectiveness and safety of anomalous coronary artery from pulmonary artery (ACAPA) patients with moderate or severe mitral valve regurgitation (MVR) receiving mitral valve plasty (MVP) concurrently. Consecutive ACAPA patients undergoing surgery between 2015 and 2021 were retrospectively included. Patients were divided into three groups: moderate MVR without MVP (non-MVP (moderate) N = 14), moderate MVR with MVP (MVP (moderate) N = 13), and severe MVR with MVP (MVP (severe) N = 13). The primary safety endpoint was in-hospital surgery-related complications. The primary effectiveness outcome was left ventricular ejection function (LVEF) and left ventricular end-diastolic diameter (LVEDD) z-score at 2- and 24-month follow-ups. Multivariable linear regression models were used to obtain the *β* coefficient. The median age of the included patients was 7.5 years (IQR 1.4–26.5). The in-hospital surgery-related complication rates were 7.1%, 15.4%, and 7.7% in non-MVP (moderate), MVP (moderate), and MVP (severe) groups, separately. At the 2-month follow-up, the non-MVP (moderate) group had a better LVEF and LVEDD z-score compared with the MVP (moderate) group (LVEF *β* = 9.22, 95%CI 1.09 to 17.35; LVEDD z-score *β* = −2.49, 95%CI −4.53 to −0.45). At the 24-month follow-up, the LVEF of all patients and the LVEDD z-score of 90% of patients in the three groups returned to normal. For ACAPA patients with moderate MVR, MVP was not necessary, especially for pediatric patients (age < 3 years) and patients with secondary MVR. Further studies for ACAPA patients with severe MVR are still needed.

## 1. Introduction

The anomalous origin of the coronary arteries from the pulmonary arteries (ACAPA) is a rare subtype of malignant coronary artery anomalies. Among them, the anomalous origin of the left coronary artery from the pulmonary artery (ALCAPA), the anomalous origin of the right coronary artery from the pulmonary artery (ARCAPA), the anomalous origin of a branch vessel (circumflex or left anterior descending artery) from the pulmonary artery, and the total anomalous origin of the coronary arteries from the pulmonary artery (TCAPA) are the four types [1]. In these types, ALCAPA is the most common congenital anomaly that usually presents as an isolated lesion, accounting for 1 in 300,000 live births (0.25% to 0.5%) [2]. ARCAPA is less frequent and is attributed to around 0.002% of all congenital heart diseases [3]. In contrast, TCAPA is extremely rare, with only a few cases reported and an unknown incidence [4]. These four types of congenital heart diseases could cause ventricular dysfunction due to myocardial ischemia.

Surgical correction to restore the two-coronary circulatory system is considered to be the standard treatment for patients with ACAPA [5]. The improvement of left ventricular function after successful correction of anomalous coronary artery origin has also been consistently demonstrated [6]. However, it has been debated whether ACAPA patients with different degrees of mitral valve regurgitation (MVR) require mitral valve plasty (MVP) concurrently with the coronary repair. Some researchers considered that mitral valve function would be improved after recovery of left ventricular (LV) function without any intervention [7,8,9], some researchers thought that moderate or severe MVR with structural abnormalities requires simultaneous management [10], and others recommended concomitant MVP at the initial operation even with a mild MVR [11].

We sought to clarify the benefit of MVP at the time of coronary repair in patients with ALCAPA and TCAPA with moderate and severe MVR. Therefore, we included ALCAPA and TCAPA patients in two centers to assess the early- and middle-term safety and effectiveness outcomes of moderate MVR patients receiving or not receiving MVP, and severe MVR patients receiving MVP.

## 2. Materials and Methods

### 2.1. Patient Population

The retrospective cohort study included 65 consecutive ACAPA patients in Beijing Fuwai Hospital and Yunnan Fuwai Hospital from 2015 to 2021. Eligible patients were congenital ALCAPA or TCAPA with moderate or severe MVR and underwent surgical repair. The diagnosis was made by Doppler echocardiography, with the addition of computed tomography angiography (CTA) or cardiac catheterization. Patients with a diagnosis of an ARCAPA (*n* = 3), no surgical treatment (*n* = 6), without MVR or mild MVR (*n* = 16), and absent MVR data (*n* = 3) were excluded. The final number of patients included in this study was 40. Informed consent was waived. This study protocol (no.2022-017-01) was approved by the local ethics committee.

**Figure 1:**
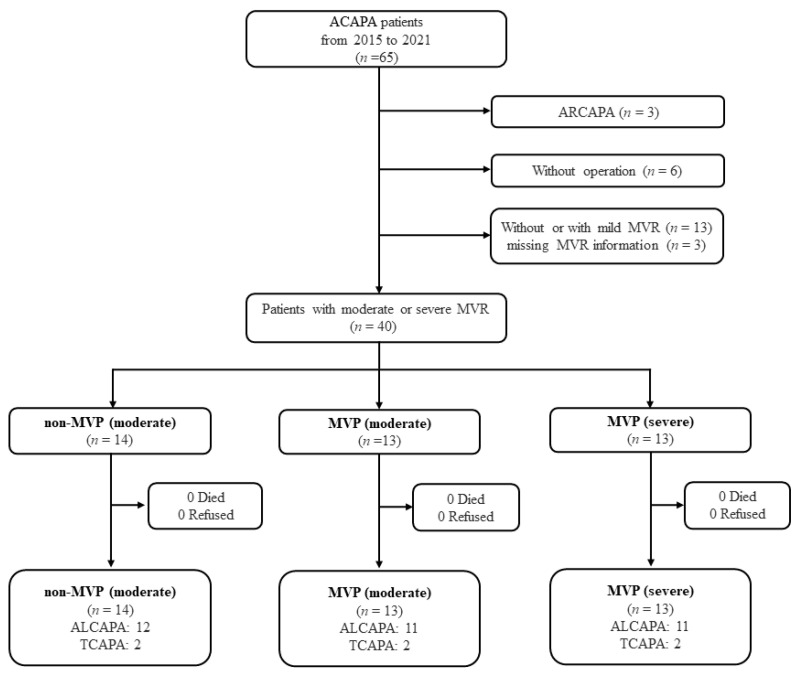
Flow chart of patient selection and follow-up. Abbreviation: ALCAPA, anomalous origin of the left coronary artery from the pulmonary artery; ARCAPA, anomalous origin of the right coronary artery from the pulmonary artery; MVR, mitral valve regurgitation; MVP, mitral valve plasty; TCAPA, total anomalous origin of the coronary arteries from the pulmonary artery.

The included patients were divided into three groups: a moderate MVR without MVP group (non-MVP (moderate)) (*n* = 14), a moderate MVR with MVP group (MVP (moderate)) (*n* = 13), and a severe MVR with MVP group (MVP (severe)) (*n* = 13) (Figure 1). MVR was evaluated according to criteria [12] (0 = none; 1 = mild; 2 = moderate; 3 = severe).

### 2.2. Variables and Outcomes

The pre-operative, intra-operative, post-operative, follow-up information and echocardiographic data were obtained from cardiac surgery databases. The pre-operative information included the age at operation, gender, body surface area (BSA), primary mitral valve regurgitation, and concomitant pre-operative cardiovascular anomalies. Primary/secondary MVR was defined according to criteria [13] (primary MVR is an intrinsic lesion of the mitral valve apparatus, and secondary MVR is an ischemic disease of the left ventricle causing tethering and maladaptation of the mitral valve leaflets). Ventricular function was assessed by standard echocardiographic methods: MVR, left ventricular ejection fraction (LVEF), and z-score of left ventricular end-diastolic dimension (LVEDD). LVEF > 50 or LVEDD z-score < 2 was considered normal heart function [14]. The echocardiographic data were analyzed by one cardiologist (Q.W.).

The primary safety outcome was surgery-related complications (which, according to clinical experience and previous literature [15], included a composite outcome of either delayed chest closure, extracorporeal membrane oxygenation (ECMO) needed, reoperation, or death during hospitalization). The primary effectiveness outcome was LVEF and LVEDD z-score at 2- and 24-month follow-ups. Secondary outcomes included intensive care unit (ICU) time and post-operative mechanical assisted ventilation time during hospitalization; LVEF, LVEDD z-score at 6- and 12-month follow-up; and MVR at the final follow-up.

### 2.3. Statistics Analysis

Continuous variables were described as mean ± SD and median (inter-quartile range (IQR)). Dichotomous variables were described as the frequency (percentage). The Kruskal–Wallis H test was used to compare the continuous variables. Fisher’s exact test was used to compare categorical data. For dichotomous outcomes, odds ratios (OR) were calculated using logistic regression models. For continuous outcomes, *β* coefficient was calculated using linear regression models. To avoid confounding bias, the models were adjusted for age and gender. Subgroup analyses for different age (<3 or ≥3 years old) groups and patients with moderate secondary MVR were conducted. Missing data were imputed using multiple imputation methods. A two-sided *p*-value < 0.05 was considered to be significant. Additionally, for the multiple comparisons, the Bonferroni correction was used, and a two-sided *p*-value < 0.025 was considered to be significant. All analyses were conducted using R (version 4.0.3) and Free Statistics software version 1.6.

## 3. Results

### 3.1. Baseline Information

Among 40 patients who underwent surgery for ACAPA, the median age at operation was 7.5 years (IQR 1.4–26.5) and 50% were female. There was no statistical difference in pre-operative cardiac function among the three groups; 15.4% and 46.2% of patients in MVP (moderate) and MVP (severe) groups had primary MVR, while none had primary MVR in the non-MVP (moderate) group. There was no difference between the three groups in terms of concomitant other cardiac malformations (Table 1 and Table S1).

### 3.2. Intra-Operative and Post-Operative Information

For the intra-operative information, there was no difference in surgical method, cardiopulmonary bypass (CPB), and cross-clamping (CCP) time among the three groups. Additionally, the time of CPB and CCP were slightly increased in the MVP (moderate) and MVP (severe) groups compared to the non-MVP (moderate) group. Detailed information on MVP is shown in Table S2.

For the post-operative information, no death occurred and no statistical difference was found for the primary safety outcome (surgery-related complications) (non-MVP (moderate): 1 (7.7%); MVP (moderate): 2 (15.4%); MVP (severe): 1 (7.7%)). However, the MVP (moderate) group had a higher risk compared with the other two groups. Three patients underwent reoperation during hospitalization, one for ECMO weaning and two for ECMO weaning combined with chest closure.

For the non-MVP (moderate) group, only one (7.7%) patient had moderate MVR, and the LVEF and LVEDD z-score were significantly better than the MVP (moderate) group (LVEF: *β* = 9.91, 95%CI, 1.38 to 21.20; LVEDD z-score: *β* = −2.04, 95%CI, −4.50 to −0.42) after adjusting for age and gender. No statistical difference was found in ICU time and ventilator time in the three groups (Table 2).

### 3.3. Follow-up Outcomes

**Figure 2:**
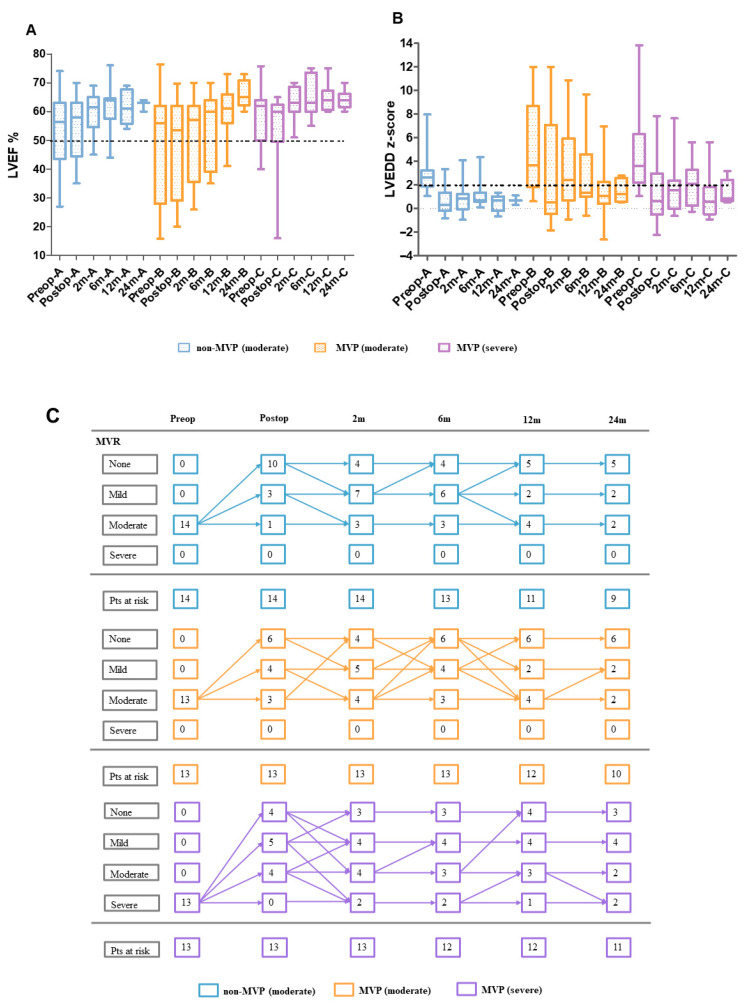
Longitudinal assessment for the primary outcome of ACAPA patients with and without mitral valve plasty. (**A**) LVEF% at pre-operation, post-operation, and follow-up. (**B**) LVEED z-score at pre-operation, post-operation, and follow-up. (**C**) Longitudinal changes in the degree of mitral valve incompetence with the corresponding number of patients at risk. Abbreviation: LVEED, left ventricular end-diastolic diameter; LVEF, left ventricular ejection fraction; MVR, mitral valve regurgitation; MVP, mitral valve plasty; Postop, post-operation; Preop, pre-operation; Pts, patients.

All patients had follow-up information, and thirty patients (75%) competed in all four follow-ups at 2 m, 6 m, 12 m, and 24 m (Figure 2). No death occurred. Two patients in the MVP (severe) group required re-operation. Both patients underwent Takeuchi operation at the initial repair and were reoperated for coronary-pulmonary artery fistula at 6 m follow-up.

For the primary effectiveness outcome, the LVEF and LVEDD z-score at 2 m follow-up of the non-MVP (moderate) group were significantly better than that of the MVP (moderate) group (LVEF: *β* = 9.22, 95%CI, 1.09 to 17.35; LVEDD z-score: *β* = −2.49, 95%CI, −4.53 to −0.45) after adjusting for age and gender (Table 2).

For the non-MVP (moderate) group, LVEF and LVEDD z-score returned to normal in all patients at 12 m follow-up; only two (22.2%) patients had moderate MVR at the last visit. For the MVP (moderate) group, LVEF returned to normal in all patients and LVEDD z-score returned to normal in 9/10 patients at 24 m follow-up; two (20.0%) patients had moderate MVR at the last visit. The cardiac function recovered faster in the non-MVP (moderate) group than in the MVP (moderate) group during the follow-up period. For the MVP (severe), LVEF returned to normal in all patients and LVEDD z-score returned to normal in 10/11 patients at 24 m follow-up; two (18.2%) patients had moderate MVR and two (18.2%) patients had severe MVR at the last visit. It is worth noting that the LVEDD z-score and MVR of the three groups all rebounded to a certain extent from discharge to 2 m follow-up (Figure 2B,C).

### 3.4. Subgroup Analysis

Subgroup analysis for patients aged <3 y and patients with moderate secondary MVR gave similar follow-up results. For patients aged <3 y, the LVEF and LVEDD z-score at 2 m and 6 m follow-up in the patients of the MVP (moderate) group were even worse compared with the MVP (moderate) group. However, for patients aged ≥3 y, no surgery-related complications happened and there was no statistical difference in the follow-up information among the three groups (Table 3 and Table 4).

### 3.5. Surgical Results of TCAPA

**Figure 3:**
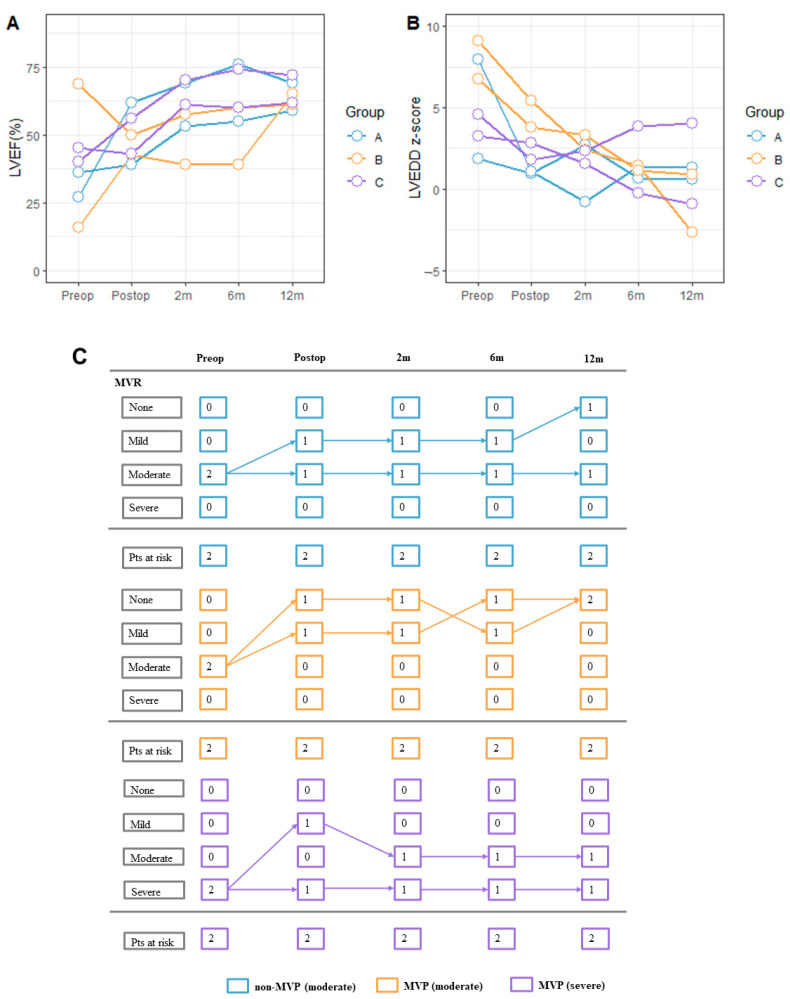
Longitudinal assessment for the primary outcome of each TCAPA patient with and without mitral valve plasty. (**A**) LVEF% of each TCAPA patient at pre-operation, post-operation, and follow-ups. (**B**) LVEED z-score of each TCAPA patient at pre-operation, post-operation, and follow-ups. (**C**) Longitudinal changes in the degree of mitral valve incompetence with the corresponding number of patients at risk. A (blue) indicated non-MVP (moderate) group, B (orange) indicated MVP (moderate) group, and C (purple) indicated MVP (severe) group. Abbreviation: LVEED, left ventricular end-diastolic diameter; LVEF, left ventricular ejection fraction; MVR, mitral valve regurgitation; Postop, post-operation; Preop, pre-operation; Pts, patients; TCAPA, total anomalous origin of the coronary arteries from the pulmonary artery.

A total of six patients were TCAPA in our study, evenly distributed among the three groups. Detailed baseline information is provided in Table S3. Two patients had surgery-related complications, one in the non-MVP (moderate) group, and the other in the MVP (moderate) group. LVEF returned to normal in all patients and LVEDD z-score returned to normal in 5/6 patients except one with severe MVR at 12 m follow-up (Figure 3).

## 4. Discussion

This study demonstrated that patients with ALCAPA and TCAPA undergoing coronary artery surgery had an improvement in LV function, without early and late death. In patients with moderate secondary MVR, simultaneous MVP did not accelerate the recovery of LV function or reduce the incidence of surgery-related complications. For patients with severe MVR, LVEF was restored 2 months after simultaneous MVP, and the size of LV was normalized in all but two cases because mitral valve function was not restored. For patients aged <3 y with moderate MVR, the benefit to recovery of cardiac function was greater when coronary repair without MVP was performed. All six patients with TCAPA underwent coronary artery reimplantation with satisfactory early and late results.

### 4.1. Moderate MVR

It is generally accepted that LV damage resulting from myocardial ischemia in ACAPA patients will cause a series of pathological changes, such as papillary muscle ischemia and ventricular dilatation and MVR frequently develops [16]. The management strategy for moderate MVR is still inconclusive. Support for not simultaneous MVP suggested that the added ischemic time associated with performing MVP in the setting of severely impaired ventricular function might do more harm than help [17]. Optimal surgical treatment of ischemic mitral regurgitation, such as Carpentier annuloplasty, was not appropriate for infants because it restricted mitral valve growth [18]. Additionally, the vast majority of MVR could be effectively improved after correction of ventricular ischemia [19]. The opposing view was that simultaneous MVP improved early post-operative cardiac ejection function and reduced post-operative mortality [11].

Our study used both LVEF and LVEED z-scores to evaluate cardiac function, because LVEF was underestimated in patients with MVP at early follow-up. Additionally, the result showed that the non-MVP group not only had a better safety outcome than the MVP group but also had better recovery of cardiac function at follow-up. Except that the non-MVP group had a slightly higher rate of moderate MVR than the MVP group at the last follow-up (28.6% vs. 23.1%). The reason might be that managed moderate MVR posed a potential hazard by increasing acute afterload on the ventricles recovering from ischemic injury [8]. Joseph et al. [7] also found ALCAPA patients with secondary MVR who did not undergo simultaneous MVP, had complete recovery of ventricular function within 6 months. According to previous studies [7,8,9,17,19] and our findings, moderate secondary MVR is usually due to chronic ischemia and could be successfully resolved by reestablishing normal coronary flow without the necessity of MVP.

### 4.2. Severe MVR

Severe MVR is generally caused by irreversible myocardial ischemic injury and papillary muscle infarction and coronary repair alone is unlikely to alleviate MVR [7]. Without simultaneous MVP, 44% of patients had no improvement of the MVR at long-term follow-up [20]. In our study, all severe MVRs were managed concurrently without early mortality and returned to normal LVEF and size of LV at 1 year post-operation. Two patients remained with severe MVR, which was caused by post-operative tendon rupture leading to prolapse of the anterior leaflet, and we will follow them regularly until adulthood before mitral valve replacement.

For severe MVR without simultaneous MVP, previous studies showed a high re-intervention rate of 37.5% and 100% [8,9]. Combined with the fact that severe MVR was a risk factor for poor prognosis [21], we, therefore, considered that patients with severe MVR undergoing simultaneous MVP might reduce early mortality and accelerate recovery of cardiac function, reducing the rate of re-intervention in the future.

### 4.3. Mitral Annuloplasty

Mitral annuloplasty has two categories: suture annuloplasty techniques and ring annuloplasty. For adult patients with ischemic MVR, the rigid or semi-rigid annuloplasty ring is preferred [22]. The suture annuloplasty techniques can be divided into the mural annulus, or commissural area according to the site. The mural annulus was managed in early clinical practice at our center, such as posterior annulus annuloplasty. Considering that posterior annuloplasty also limited ring growth, there was a risk of stenosis in the long term [23]. In contrast, management of the commissural area was simple, not time-consuming, and effective in reducing regurgitation while having less impact on the growth of the annulus [24]. Therefore, simultaneous mitral annuloplasty at the bilateral commissural area was a logical approach for improving cardiac output during the critical post-operative period.

### 4.4. Treatment of TCAPA

In patients with TCAPA, angiography or CT angiography should be considered the gold standard for diagnosis, and echocardiographic evaluation is helpful as an aid to diagnosis [25]. Numerous cases of TCAPA were incorrectly diagnosed as ALCAPA based on pre-operative echocardiogram, which may be related to the inherent difficulty assessing the right coronary artery on echocardiography [26]. Additionally, pre-operative misdiagnosis would, in turn, lead to intraoperative devastating consequences. The two main treatment strategies that were used in patients with TCAPA were aortic reimplantation technique or Takeuchi repair using intrapulmonary baffle, and a meta-analysis showed that 18/27 of patients had a single coronary lesion, 14/27 used the aortic reimplantation technique, and 4/27 had simultaneous MVP [4]. In our study, six patients with TCAPA underwent aortic reimplantation and four patients had simultaneous MVP. No early death occurred and one patient received ECMO assistance due to intraoperative difficulties in weaning from extracorporeal circulation and was successfully weaning after 7 days. However, the lack of standardized, large sample-size clinical studies prevented an objective comparison of clinical techniques for TCAPA. The LV and mitral valve conditions of TCAPA are similar to those of ALCAPA, but age, symptoms, and pathophysiology vary from ALCAPA [27]. The management strategy for MVR during the restoration of the two-coronary circulatory system should be considered an aggressive intervention for mitral valves with structural abnormalities. Each cardiac center should carefully design an individualized treatment strategy for TCAPA.

### 4.5. Strength and Limitations

This study analyzed the cardiac function and MVR at four follow-ups in ALCAPA and TCAPA patients with or without MVP grouped by baseline MVR degree and had an adequate adjustment for possible confounding factors. However, this study still had several limitations, such as the retrospective study design and the relatively short average follow-up period of 24 months. In addition, all severe MVR was repaired with MVP concurrently at our center, so we could not compare the results of severe regurgitation untreated in this study, but we considered the findings of previous studies. Finally, our study only relies on Doppler echocardiography to evaluate cardiac function, which may not be enough to accurately determine the actual situation of LV contractility. In future research, we will continue to follow up on the results of magnetic resonance imaging to verify the recovery of cardiac function.

## 5. Conclusions

In conclusion, patients with ACAPA could obtain a good prognosis through reconstruction of double coronary blood supply. In our experience, MVP was not necessary for ACAPA patients with moderate MVR, especially for pediatric patients (age < 3 y) and patients with secondary MVR, since secondary MVR is usually due to chronic ischemia and could be effectively improved by restoring normal coronary blood flow. Further studies for ACAPA patients with severe MVR and TCAPA patients are still needed.

## Figures and Tables

**Table 1:** Baseline characteristics of ACAPA patients with and without mitral valve plasty.

Variables	Non-MVP (Moderate) (*n* = 14)	MVP (Moderate) (*n* = 13)	MVP (Severe) (*n* = 13)	*p* Value
Pre-operation				
Age (months)	25.5 ± 22.3 24.5 (1.3, 45.0)	11.8 ± 17.0 4.6 (1.5, 10.0)	13.1 ± 16.2 5.9 (1.3, 21.0)	0.124
Female	9 (64.3)	6 (46.2)	5 (38.5)	0.384
BSA (m^2^)	1.2 ± 0.6 1.5 (0.5, 1.6)	0.9 ± 0.5 0.7 (0.5, 1.3)	1.0 ± 0.6 0.7 (0.5, 1.6)	0.296
Type				0.996
TCAPA	2 (14.3)	2 (15.4)	2 (15.4)	
ALCAPA	12 (85.7)	11 (84.6)	11 (84.6)	
Cardiac function decrease	6 (42.9)	5 (38.5)	2 (15.4)	0.268
LVEF (%)	53.3 ± 13.5 56.5 (46.0, 63.0)	47.8 ± 20.7 56.0 (28.0, 61.0)	58.4 ± 10.7 62.0 (55.0, 63.0)	0.389
LVEDD z-score	3.0 ± 1.7 2.6 (1.9, 3.2)	5.4 ± 3.9 3.6 (1.9, 8.2)	4.6 ± 3.7 3.6 (2.3, 5.9)	0.160
Primary MVR	0 (0.0)	2 (15.4)	6 (46.2)	0.006
PAH	2 (14.3)	0 (0.0)	3 (23.1)	0.199
Ventricular wall aneurysm	0 (0.0)	1 (7.7)	1 (7.7)	0.567
Concomitant cardiovascular anomaly ^a^	2 (14.3)	2 (15.4)	2 (15.4)	0.996
Intra-operation				
Surgical method				0.810
Direct reimplantation	12 (85.7)	11 (84.6)	11 (84.6)	
Takeuchi operation	2 (14.3)	2 (15.4)	2 (15.4)	
CPB (min)	135.6 ± 66.7 112.5 (71.0, 158.0)	137.5 ± 41.2 123.0 (109.0, 153.0)	176.4 ± 92.9 145.0 (125.0, 185.0)	0.254
CCP (min)	84.7 ± 38.6 77.5 (50.0, 110.0)	91.2 ± 33.5 81.0 (69.0, 116.0)	112.3 ± 42.0 109.0 (82.0, 132.0)	0.238

^a^ Concomitant cardiovascular anomaly: patient with PAS, PDA, VSD, ASD, PFO, or aorta overriding. Abbreviation: ACAPA, anomalous coronary artery from the pulmonary artery; ALCAPA, anomalous left coronary artery from the pulmonary artery; ASD, atrial septal defect; BSA, body surface area; CCP, cross-clamping; CPB, cardiopulmonary bypass; LVEDD, left ventricular end-diastolic diameter; LVEF, left ventricular ejection fraction; MVR, mitral valve regurgitation; MVP, mitral valve plasty; PAH, pulmonary artery hypertension; PAS, pulmonary artery stenosis; PDA, patent ductus arteriosus; PFO, patent foramen ovale; TCAPA, total anomalous origin of the coronary arteries from the pulmonary artery; VSD, ventricular septal defect.

**Table 2:** Post-operative and follow-up information of ACAPA patients with and without mitral valve plasty.

Variables	Non-MVP (Moderate) (*n* = 14)	MVP (Moderate) (*n* = 13)	MVP (Severe) (*n* = 13)	*p* Value	*β*/OR (95%CI) ^a^
					Non-MVP (Moderate) vs. MVP (Moderate)
Post-operation					
ICU time (d)	7.3 ± 14.1 1.5 (1.0, 4.0)	4.8 ± 5.3 2.0 (1.0, 7.0)	7.3 ± 12.9 2.0 (1.0, 4.0)	0.822	6.79 (−2.10, 15.69) *p* = 0.144
Duration of ventilation (min)	98.7 ± 214.9 17.5 (8.0, 23.0)	40.8 ± 54.6 15.0 (11.0, 36.0)	86.8 ± 178.0 17.0 (11.0, 25.0)	0.639	118.17 (−11.36, 247.70) *p* = 0.083
Cardiac function decrease	5 (35.7)	7 (53.8)	3 (23.1)	0.265	0.27 (0.04, 2.03) *p* = 0.204
LVEF (%)	55.1 ± 10.8 58.0 (45.0, 62.0)	48.0 ± 16.6 53.6 (30.0, 59.0)	53.8 ± 14.2 60.0 (56.0, 60.0)	0.388	9.91 (1.38, 21.20) *p* = 0.045
LVEDD z-score	0.7 ± 1.2 0.3 (−0.2, 1.1)	3.1 ± 4.5 0.5 (−0.4, 6.7)	1.4 ± 2.9 0.5 (−0.5, 2.3)	0.763	−2.04 (−4.50, −0.42) *p* = 0.033
MVR	1 (7.1)	2 (15.4)	4 (30.8)	0.264	0.43 (0.02, 11.58) *p* = 0.615
Surgery-related complications *	1 (7.1)	2 (15.4)	1 (7.7)	0.733	0.43 (0.02, 9.58) *p* = 0.435
Delayed chest closure	1 (7.1)	1 (7.7)	0 (0.0)	0.329	NA
ECMO needed	1 (7.1)	1 (7.7)	1 (7.7)	0.998	NA
Reoperation	1 (7.1)	1 (7.7)	1 (7.7)	0.998	NA
Death	0 (0.0)	0 (0.0)	0 (0.0)	NA	NA
Follow-up					
2-month follow-up					
LVEF%	59.6 ± 6.9 61.5 (55.0, 65.0)	50.7 ± 14.6 57.0 (39.0, 60.0)	63.0 ± 5.5 63.0 (60.0, 68.0)	0.040	9.22 (1.09, 17.35) *p* = 0.033
LVEDD z-score	0.8 ± 1.4 0.8 (0.2, 1.2)	3.3 ± 3.4 2.4 (1.0, 5.2)	1.8 ± 2.4 1.6 (0.1, 2.4)	0.044	−2.49 (−4.53, −0.45) *p* = 0.023
6-month follow-up					
LVEF%	61.7 ± 8.6 64.0 (60.0, 64.0)	52.8 ± 12.8 60.0 (39.0, 64.0)	65.3 ± 7.1 63.0 (60.0, 73.0)	0.088	8.97 (−1.63, 19.57) *p* = 0.111
LVEDD z-score	1.2 ± 1.2 0.7 (0.6, 1.3)	2.6 ± 3.0 1.3 (1.0, 4.6)	1.9 ± 2.0 2.1 (0.6, 2.7)	0.394	−0.86 (−3.10, 1.38) *p* = 0.457
12-month follow-up					
LVEF%	61.4 ± 5.8 61.0 (56.5, 66.5)	60.8 ± 9.5 61.0 (56.0, 66.0)	64.8 ± 4.9 64.0 (61.0, 66.5)	0.497	−0.26 (−8.36, 7.84) *p* = 0.95
LVEDD z-score	0.5 ± 0.7 0.7 (−0.0, 1.0)	1.5 ± 2.4 1.1 (0.4, 2.2)	1.2 ± 2.2 0.6 (−0.5, 1.8)	0.558	−0.13 (−2.07, 1.80) *p* = 0.893
24-month follow-up					
LVEF%	62.3 ± 2.1 63.0 (60.0, 64.0)	66.0 ± 4.7 65.0 (63.0, 70.0)	64.2 ± 3.4 64.0 (62.0, 65.0)	0.416	−5.48 (−11.16, 0.19) *p* = 0.091
LVEDD z-score	0.7 ± 0.6 0.7 (0.3, 1.1)	1.5 ± 1.1 1.2 (0.6, 2.3)	1.4 ± 1.1 0.8 (0.8, 1.7)	0.680	−0.29 (−3.48, 2.91) *p* = 0.866

* Surgery-related complications that included delayed chest closure, ECMO needed, reoperation, and death. ^a^ The models were adjusted for age and gender. Abbreviation: ACAPA, anomalous coronary artery from the pulmonary artery; CPB, cardiopulmonary bypass; CCP, cross-clamping; ICU, intensive care unit; ECMO, extracorporeal membrane oxygenation; LVEDD, left ventricular end-diastolic diameter; LVEF, Left ventricular ejection fraction; MVR, mitral valve regurgitation; MVP, mitral valve plasty.

**Table 3:** Post-operative and follow-up information of ACAPA patients with and without mitral valve plasty grouped by age.

Variables	Age < 3 y				Age ≥ 3 y			
	Non-MVP (Moderate) (*n* = 4)	MVP (Moderate) (*n* = 5)	MVP (Severe) (*n* = 6)	*p* Value ^a^	Non-MVP (Moderate) (*n* = 10)	MVP (Moderate) (*n* = 8)	MVP (Severe) (*n* = 7)	*p* Value
Post-operation								
Surgery-related complications *	1 (25.0)	2 (40.0)	1 (16.7)	0.682	0 (0.0)	0 (0.0)	0 (0.0)	NA
Follow-up								
2-month follow-up								
LVEF%	63.8 ± 7.4 66.5 (59.0, 68.0)	34.2 ± 6.3 32.0 (32.0, 39.0)	63.0 ± 7.0 64.0 (60.0, 69.0)	<0.0001	57.9 ± 6.3 60.0 (55.0, 62.0)	61.0 ± 5.1 60.0 (57.3, 64.0)	63.0 ± 4.4 61.0 (60.0, 68.0)	0.178
LVEDD z-score	0.0 ± 1.8 −0.8 (−0.9, 1.0)	6.5 ± 3.1 6.7 (5.2, 7.4)	3.2 ± 2.9 2.4 (1.8, 5.6)	0.014	1.1 ± 1.1 1.1 (0.5, 1.2)	1.3 ± 1.5 1.0 (0.3, 2.8)	0.5 ± 0.9 0.3 (−0.2, 1.6)	0.442
6-month follow-up								
LVEF%	65.0 ± 10.5 64.0 (55.0, 76.0)	40.4 ± 6.2 39.0 (37.0, 40.0)	66.0 ± 10.0 67.0 (57.5, 74.0)	0.003	60.0 ± 8.0 63.5 (60.0, 64.0)	63.2 ± 3.8 62.5 (60.0, 64.0)	64.8 ± 4.9 63.0 (63.0, 65.0)	0.411
LVEDD z-score	0.9 ± 0.4 0.7 (0.6, 1.4)	4.9 ± 3.0 4.6 (4.3, 4.6)	3.5 ± 1.6 3.3 (2.4, 4.7)	0.088	1.4 ± 1.5 1.0 (0.6, 1.3)	0.7 ± 0.8 1.0 (0.1, 1.1)	0.6 ± 1.0 0.6 (−0.1, 0.6)	0.460
12-month follow-up								
LVEF%	64.0 ± 5.0 64.0 (59.0, 69.0)	54.4 ± 9.3 56.0 (50.0, 60.0)	65.0 ± 8.7 60.0 (60.0, 75.0)	0.191	59.8 ± 6.2 58.0 (55.0, 63.0)	66.2 ± 5.9 65.5 (61.0, 73.0)	64.6 ± 2.3 65.0 (63.0, 65.0)	0.153
LVEDD z-score	0.9 ± 0.4 0.8 (0.6, 1.3)	2.3 ± 3.5 2.2 (1.1, 3.9)	3.1 ± 2.2 1.8 (1.8, 5.6)	0.637	0.3 ± 0.8 0.3 (−0.4, 0.9)	0.9 ± 0.8 0.7 (0.4, 1.3)	−0.2 ± 0.6 −0.3 (−0.7, 0.3)	0.129
24-month follow-up								
LVEF%	64.0 ± 0.1 64.0 (64.0, 64.0)	62.7 ± 2.5 63.0 (60.0, 65.0)	65.0 ± 5.0 65.0 (60.0, 70.0)	0.782	61.5 ± 2.1 61.5 (60.0, 63.0)	69.3 ± 4.0 70.0 (65.0, 73.0)	63.3 ± 1.5 63.0 (62.0, 65.0)	0.058
LVEDD z-score	1.1 ± 0.1 1.1 (1.1, 1.1)	1.7 ± 1.6 1.7 (0.6, 2.8)	1.8 ± 1.3 1.7 (0.5, 3.2)	0.954	0.3 ± 0.1 0.3 (0.3, 0.3)	1.2 ± 1.0 1.2 (0.5, 1.9)	0.8 ± 0.1 0.8 (0.8, 0.8)	0.368

* Surgery-related complications that included delayed chest closure, ECMO needed, reoperation, and death. ^a^ A two-sided *p*-value < 0.025 was considered to be significant. Abbreviation: ACAPA, anomalous coronary artery from the pulmonary artery; ECMO, extracorporeal membrane oxygenation; LVEDD, left ventricular end-diastolic diameter; LVEF, left ventricular ejection fraction; MVP, mitral valve plasty.

**Table 4:** Post-operative and follow-up information of ACAPA patients with moderate secondary MVR.

Variables	Non-MVP (Moderate) (*n* = 14)	MVP (Moderate) (*n* = 11)	*β* (95%CI) ^a^
			Non-MVP (Moderate) vs. MVP (Moderate)
Post-operation			
Surgery-related complications *	1 (7.1)	1 (9.1)	NA
Follow-up			
2-month follow-up			
LVEF%	59.6 ± 6.9 61.5 (55.0, 65)	51.5 ± 14.5 57.0 (39.0, 64)	8.73 (0.77, 16.70) 0.039
LVEDD z-score	0.8 ± 1.4 0.8 (0.2, 1.2)	3.0 ± 3.5 2.4 (0.4, 5.2)	−2.30 (−4.38, −0.21) 0.039
6-month follow-up			
LVEF%	61.7 ± 8.6 64.0 (60.0, 64)	53.4 ± 13.3 60.0 (39.0, 64)	8.73 (−2.01, 19.46) 0.126
LVEDD z-score	1.2 ± 1.2 0.7 (0.6, 1.3)	2.6 ± 3.2 1.3 (1.0, 4.3)	−0.88 (−3.19, 1.43) 0.466
12-month follow-up			
LVEF%	61.4 ± 5.8 61.0 (56.5, 66)	62.2 ± 9.7 65.0 (60.0, 66)	−1.06 (−9.09, 6.98) 0.799
LVEDD z-score	0.5 ± 0.7 0.7 (−0.0, 1.0)	1.4 ± 2.6 1.1 (0.4, 2.2)	−0.06 (−2.00, 1.89) 0.953
24-month follow-up			
LVEF%	62.3 ± 2.1 63.0 (60.0, 64)	67.8 ± 4.6 67.5 (64.0, 71)	−6.06 (−11.77, −0.34) 0.046
LVEDD z-score	0.7 ± 0.6 0.7 (0.3, 1.1)	1.2 ± 0.9 1.2 (0.6, 1.9)	0.18 (−2.96, 3.32) 0.916

* Surgery-related complications that included delayed chest closure, ECMO needed, reoperation, and death. ^a^ The models were adjusted for age and gender. Abbreviation: ACAPA, anomalous coronary artery from the pulmonary artery; ECMO, extracorporeal membrane oxygenation; LVEDD, left ventricular end-diastolic diameter; LVEF, left ventricular ejection fraction; MVP, mitral valve plasty.

## Data Availability

The data will be shared on reasonable request to the corresponding author.

## Supplementary Materials

The following supporting information can be downloaded at: https://www.mdpi.com/article/10.3390/jcdd10020075/s1, Supplementary information: Surgical procedures of direct reimplantation, Takeuchi operation, and mitral valve plasty; Table S1: Baseline characteristics of ACAPA patients with and without mitral valve plasty; Table S2: MV pathology, MVR grade pre-operatively, operative techniques of MVP, and MVR grade post-operatively in all 26 patients; Table S3: Detailed baseline information of TCAPA patients.
