# Supplementary material for: Effectiveness and Safety of Mitral Valve Plasty in Patients with an Anomalous Origin of the Coronary Artery from the Pulmonary Artery
Source: J Cardiovasc Dev Dis. 2023 Feb 9;10(2):75. doi: 10.3390/jcdd10020075 (PMC9959487; doi:10.3390/jcdd10020075)
Supplement: Supplementary file 1 [file jcdd-10-00075-s001.zip › jcdd-2145766-supplementary.pdf]

Effectiveness and Safety of Mitral Valve Plasty in Patients with an Anomalous Origin of the Coronary Artery from the Pulmonary Artery

(Supplementary Materials)

Supplementary information. Surgical procedures of Direct reimplantation, Takeuchi operation, and mitral valve plasty.

Table S1. Baseline characteristics of ACAPA patients with and without mitral valve plasty.

Table S2. Mitral valve pathology, MVR grade preoperatively, operative techniques of MVP, and MVR grade postoperatively in all 26 patients.

Table S3. Detailed baseline information of TCAPA patients.

Surgical Procedures

All patients underwent median sternotomy, bicaval cannulation, and hypothermic cardiopulmonary bypass. Thirty-four patients (85%) through the aortic root and six patients (15%) through the aortic root and main pulmonary artery (PA), all of whom were perfused with crystalloid cardiac arrest solution to induce cardiac arrest. The pulmonary aorta (PA) was transected proximal to the bifurcation and the location of the anomalous opening was determined. Depending on the anatomical location of the anomalous coronary artery ostium, direct reimplantation was preferred when the coronary ostium arose from the right facing or anterior sinus of Valsalva of the pulmonary artery, while Takeuchi operation was more often performed when the anomalous coronary ostium arose from the non facing pulmonary sinus. In our center, anomalous coronary artery treatment was accomplished mainly by direct aortic reimplantation (34 cases) or tunneling (6 cases).

Direct reimplantation. Twenty-eight (82%) left coronary arteries, three (8.8%) left and right coronary arteries, and three (8.8%) anomalous single coronary arteries were removed in button-shaped cuffs and subsequently mobilized. The aorta was opened transversely to create an opening for the left coronary sinus in 28 patients, the left and right coronary sinuses in 3 patients, and the right coronary sinus in 3 patients. The coronary button was tension-free anastomosed to the aorta from within the aortic lumen through 7-0 absorbable continuous sutures. The pulmonary artery trunk was reconstructed with an autologous pericardial patch and PA continuity was restored by direct end-to-end anastomosis.

Takeuchi operation. A hole was made in the right wall of the main PA, opening the aorta obliquely and creating an aortopulmonary window in the left wall of the aorta with a 4- or 4.5- mm puncher, taking care to avoid damaging the aortic valve leaflets. Both holes were approached with 5-0 absorbable sutures. A coronary tunnel was then created with a bovine pericardial patch for the left coronary artery in six cases, approximating the posterior wall of the main PA and along the nadir of the Valsalva sinus back to the aortopulmonary window. The anterior defect of the main PA was covered with autologous pericardium.

mitral valvuloplasty. Twenty-six patients underwent simultaneous mitral valve plasty (MVP). (Details in **Table S2**).

Table S1. Baseline characteristics of ACAPA patients with and without mitral valve repair

Variables	non-MVP (moderate) (n=10)	MVP (moderate) (n=13)	MVP (severe) (n=13)	*P* value
Concomitant cardiovascular anomaly				
PAS	0 (0.0)	1 (7.7)	0 (0.0)	0.345
PDA	0 (0.0)	1 (7.7)	0 (0.0)	0.345
VSD	0 (0.0)	1 (7.7)	0 (0.0)	0.345
ASD	0 (0.0)	2 (15.4)	0 (0.0)	0.112
PFO	2 (14.3)	0 (0.0)	2 (15.4)	0.342
Overriding aorta	0 (0.0)	1 (7.7)	0 (0.0)	0.345

Abbreviation: ASD, atrial septal defect; PAS, Pulmonary artery stenosis; PDA, Patent ductus arteriosus; PFO, patent foramen ovale; PVS, Pulmonary valve stenosis; VSD, Ventricular septal defect.

Table S2 MV pathology, MR grade preoperatively, operative techniques of MVP, and MR grade postoperatively in all 26 patients

Patient	pre-operation MVR grade	age (year)	MV pathology	MV repair technique	MR grade at last visit
1	Moderate	1.5	Chorda tendineae fibrosis; Ring dilatation	Bilateral commissuroplasty	mild
2	Moderate	2.7	Ring dilatation	Posterior annuloplasty	moderate
3	Moderate	26.0	Mitral valve leaflet thickening; Ring dilatation	Ring annuloplasty	none
4	Moderate	0.5	Chorda tendineae fibrosis; Papillary muscle fibrosis; Ring dilatation	Bilateral commissuroplasty	none
5	Moderate	0.5	Chorda tendineae fibrosis; Prolapse of anterior and posterior leaflet; Anterior leaflet cleft	Posterior annuloplasty; Triangular resection of anterior leaflet	none
6	Moderate	6.0	Chorda tendineae fibrosis; Papillary muscle fibrosis; Ring dilatation	Posterior annuloplasty; release of papillary muscle	moderate
7	Moderate	4.6	Ring dilatation; Chorda tendineae fibrosis	Bilateral commissuroplasty	moderate
8	Moderate	1.5	Chorda tendineae fibrosis; Ring dilatation	Posterior annuloplasty	none
9	Moderate	60.0	Ring dilatation	Ring annuloplasty	mild
10	Moderate	3.8	Chorda tendineae fibrosis; Prolapse of anterior and posterior leaflet	Annular reduction sutures; Triangular resection of anterior leaflet	mild
11	Moderate	9.0	Ring dilatation	Posterior annuloplasty	none
12	Moderate	27.0	Ring dilatation	Ring annuloplasty	none
13	Moderate	10.0	Papillary muscle fibrosis; Ring dilatation	Bilateral commissuroplasty	mild

14	Severe	42.0	Chorda tendineae fibrosis; Papillary muscle fibrosis; Mitral valve leaflet thickening	Ring annuloplasty	none
15	Severe	24.0	Ring dilatation	Ring annuloplasty	none
16	Severe	1.3	Prolapse of anterior and posterior leaflet; Mitral valve leaflet thickening; Chorda tendineae fibrosis; Papillary muscle fibrosis	Bilateral commissuroplasty; release of papillary muscle	mild
17	Severe	2.4	Prolapse of anterior leaflet; Ring dilatation	Annular reduction sutures	severe
18	Severe	0.3	Ring dilatation; Papillary muscle fibrosis	Posterior annuloplasty	mild
19	Severe	0.7	Chorda tendineae fibrosis; Prolapse of anterior leaflet; Ring dilatation	Bilateral commissuroplasty	mild
20	Severe	2.9	Chorda tendineae fibrosis; Papillary muscle fibrosis; Prolapse of anterior leaflet; Ring dilatation	Bilateral commissuroplasty	moderate
21	Severe	5.9	Chorda tendineae fibrosis; Papillary muscle fibrosis; Mitral valve leaflet thickening	Bilateral commissuroplasty	mild
22	Severe	8.0	Chorda tendineae fibrosis; Papillary muscle fibrosis; Prolapse of anterior leaflet; Ring dilatation	Posterior annuloplasty; release of papillary muscle	moderate
23	Severe	21.0	Ring dilatation; Mitral valve leaflet thickening	Ring annuloplasty	mild
24	Severe	48.0	Mitral valve leaflet thickening; Ring dilatation	Ring annuloplasty	none
25	Severe	13.0	Chorda tendineae fibrosis; Papillary muscle fibrosis; Prolapse of posterior leaflet	Posterior annuloplasty; Shortening of leaflet chords	none
26	Severe	0.2	Ring dilatation; Mitral valve leaflet thickening; Chorda tendineae fibrosis; Papillary muscle fibrosis; Anterior leaflet cleft	Posterior annuloplasty; release of papillary muscle; shortening of leaflet chord	severe

Abbreviation: MVR: mitral valve regurgitation; MV: mitral valve.

Table S3 Detailed baseline information of TCAPA patients

No	Age (year)	Gender	Symptoms	LVEF	MVR	TVR	Concomitant cardiovascular anomaly	Single coronary artery anomaly	Origin	The course between the Ao and Po
1	8.0	Female	Shortness of breath for 2 years	45	Severe	None	None	No	From right and left cusp	Without
2	9.0	Female	Lips purple attack when crying	68.7	Moderate	Severe	TOF	Yes	From right cusp	Without
3	1.3	Female	Physical examination found	36	Moderate	None	None	No	From left cusp	With
4	0.3	Female	Intermittent shortness of breath	41	Moderate	Severe	ASD	Yes	From right cusp	With
5	1.5	male	Disgust activity, sweaty	20	Moderate	None	None	No	From right cusp	With
6	0.2	Female	Shortness of breath	71	Severe	None	PFO	Yes	From the right pulmonary artery	With course along one side of Po

Abbreviation: Ao, aorta; ASD, atrial septal defect; LVEF, left ventricular ejection fraction; MVR, mitral valve regurgitation; Po, pulmonary artery; PFO, patent foramen ovale; TOF, Tetralogy of Fallot; TVR, tricuspid valve regurgitation.
